# Transfer of Multidrug-Resistant Bacteria between Intermingled Ecological Niches: The Interface between Humans, Animals and the Environment

**DOI:** 10.3390/ijerph10010278

**Published:** 2013-01-14

**Authors:** Paulo Martins da Costa, Luís Loureiro, Augusto J. F. Matos

**Affiliations:** 1 ICBAS—Abel Salazar Biomedical Sciences Institute, University of Porto, Porto, 4050-313, Portugal; E-Mail: ajmatos@icbas.up.pt; 2 CIIMAR—Interdisciplinary Center for Marine and Environmental Research, University of Porto, Porto, 4050-123, Portugal; 3 Tulane University School of Public Health and Tropical Medicine, New Orleans, LA 70112, USA; E-Mail: lfljloureiro@gmail.com; 4 Multidisciplinary Unit for Biomedical Research (UMIB), University of Porto, Porto, 4050-313, Portugal

**Keywords:** antimicrobials, resistance, humans, animals, environment

## Abstract

The use of antimicrobial agents has been claimed to be the driving force for the emergence and spread of microbial resistance. However, several studies have reported the presence of multidrug-resistant bacteria in populations exposed to low levels of antimicrobial drugs or even never exposed. For many pathogens, especially those organisms for which asymptomatic colonization typically precedes infection (e.g., *Enterococcus* spp. and *Escherichia coli*), the selective effects of antimicrobial use can only be understood if we considerer all biological and environmental pathways which enable these bacteria, and the genes they carry, to spread between different biomes. This ecological framework provides an essential perspective for formulating antimicrobial use policies, precisely because it encompasses the root causes of these problems rather than merely their consequences.

## 1. Introduction

Antimicrobial resistance remains a serious global health concern, and solutions to address this fact are urgently required. This is not only the case in developed countries, where there are several policies governing the use of these drugs, and capacity (both human and capital) exists to assess the levels of antimicrobial resistance. In developing countries, where the health and related sectors are challenged with various constraints (such as access to better or new drugs when resistance is suspected) or even when the priority is to provide basic health care, certainly strategies to control and prevent resistance are not at the top list of priorities. The phenomenon of microbial resistance, which is based on genetic plasticity of bacteria, has emerged as a consequence of the selective pressure exerted by the antimicrobial usage in human medicine, veterinary medicine, animal production, fish production, agriculture and food technology [1,2,3,4]. Antimicrobial resistance is exacerbated due to over-prescription of antibiotics and increased use in human and animal medicine, as a consequence of the growing number of invasive medical procedures and the enormous increase in the number of immunocompromised individuals and patients with chronic debilitating diseases [5,6]. The increasing mobility of people and food products, as well as the absence of environmental barriers between different living communities, raised the risk of the spread of antimicrobial resistance worldwide [7,8,9].

Whenever antimicrobials are used, bacteria inevitably develop resistance mechanisms either through spontaneous mutations or by acquiring genes from other bacteria. The later may occur by transduction (mediated by bacteriophages); conjugation (which involves direct cell-to-cell contact and transfer of plasmids or transposons); or transformation, involving the uptake of free DNA that results from bacterial lysis [10,11]. Horizontal transfer of genetic elements between bacteria is critical to the dissemination of resistance, particularly within a mixed bacterial population (e.g., intestine, respiratory mucosa and skin) in the presence of antimicrobial drugs [12,13]. The co-existence of various resistance genes in the same plasmid or transposon results in the incidental transfer of the whole group, even if the selective pressure is directed towards a specific gene [14]. This co-selection mechanism impacts the establishment of a linear relationship between the use of a specific antibiotic and the emergence of the corresponding resistance [15]. The fact that the recipient cell receives all the genetic competences mediated by a certain plasmid may result in more complex consequences, such as the transfer of virulence determinants under the selective pressure imposed by the presence of antibiotics or, in opposition, the non-selected transmission of antimicrobial resistance genes driven by the presence of heavy metals or disinfectants [16,17]. This dynamic also favours the optimization of these genetic elements, dashing initial hopes of reversing resistance by simply reducing antibiotic use [6,18].

The probability of premature death in humans due to infection would be 40% higher if antimicrobials were non-existent [19]. In spite of remarkable technological and scientific advances in the area of molecular genetics, a significant decline in the development of new antibiotics was evident from the 1970s [20,21]. In opposition, an emergence of multidrug-resistant strains created an atmosphere of anxiety that called for immediate action. Therapeutic failures associated to antimicrobial resistance increases morbidity and mortality, with serious implications at individual, social and economical levels. Furthermore, antimicrobial resistance limits the choice of therapeutic agents and increases the potential for treatment failures and adverse clinical outcomes [22].

Besides these clinical consequences, equally serious ecological and epidemiological effects resulted from antimicrobial resistance [23], which includes the paradoxical increased vulnerability to infection among individuals receiving antimicrobial therapy for unrelated reasons. These “excess cases” are caused by the combination of the enrichment of antimicrobial-resistant pathogens with the increase in vulnerability to infection, due to the inhibition of the commensal microbiota of the skin, respiratory and digestive mucosae, which exert a protective effect against colonization and infection by exogenous organisms [14,24]. In 1962, Bohnhoff and Miller [25] demonstrated that the partial inhibition of enteric flora in mice treated with streptomycin administration, reduced up to 100,000 times the infective dose of streptomycin-resistant *Salmonella* when compared with mice having an undisturbed intestinal flora. More recently, Barza and Travers [26] estimated that 13 to 26% of non-typhoidal *Salmonella* infections in humans could be attributed to the unrelated administration of antibiotics (e.g., for the treatment of tonsillitis) to asymptomatic carriers of resistant *Salmonella* strains. Physicians should be aware that patients who are taking antimicrobial agents for any reason are at increased risk for acquiring antimicrobial-resistant bacteria. From an epidemiological point of view, the massive enrichment of resistant strains increases the probability of direct infection by inter-related individuals (e.g., hospitalized patients and their relatives) and indirect dissemination via environmental pathways [26,27,28].

## 2. Antimicrobial Use in Animals

**Figure 1:**
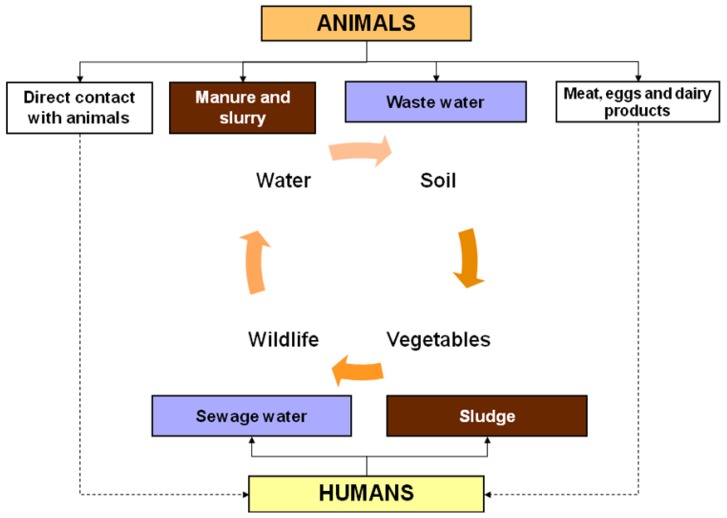
Conceptual model of transfer of drug-resistant bacteria between the human population, the animal population and the environment.

In the past, an anthropocentric view of human pathogens led us to ignore for decades the existence of an ecological cycle that does not directly involve humans. As in human medicine, the use of antimicrobial drugs in veterinary medicine creates a selective pressure for the emergence of antimicrobial-resistant bacteria, including animal pathogens, human pathogens that have animal reservoirs, and commensal bacteria that are present in animals [29,30]. Available data indicates that the use of antimicrobial agents in animals—including the use of drugs that are critically important to human medicine—is considerable and may even exceed their use in human medicine [31]. Drug-resistant bacteria selected by this selective pressure can spread to humans either by the food supply (e.g., meat, fish, eggs and dairy products), direct contact with animals or, more indirectly, through environmental pathways (Figure 1) [32,33]. These bacteria are then able to colonize or, at least, transfer genes conferring antibiotic resistance to pathogenic and commensal bacteria of humans, as firstly reported by Smith in 1969 [34]. Taking an antimicrobial agent decreases the infectious dose for a pathogen that is already resistant (due to suppression of the normal protective flora that is susceptible to that antimicrobial agent) and, simultaneously, increases the likelihood that native host flora will acquire resistance from ingested resistant bacteria [33].

### 2.1. Companion Animals

During the last decade, there has been an increasing awareness of the potential problems that the selection of antimicrobial-resistant bacteria among companion animals may cause on human health. This is due in part to the increasing prescription for pets of antimicrobial substances that are critical to human medicine, but also due to the close contact between pets and their human co-habitants. The growing number of household pets and their increasing health care standards led to an augmented number of geriatric animals, which have an extensive medical history, including antimicrobial drug administration, and longer contact with owners, increasing both the risk of antimicrobial resistance emergence and inter-species clonal spread.

Taking into account some frequent behaviours of dogs and cats inside and outside the household (e.g., grooming, perigenital and skin licking, rolling on faeces and coprophagy), it is expectable that contamination of their hair, skin and mouth with faecal and body surface bacteria. Hence, subsequent spread of these bacteria to human co-habitants can occur directly, by skin to skin contact and contact with bacteria in the saliva or faeces, or indirectly, via the household environment [35]. Data from a recent study revealed the presence of the same multidrug-resistant *Escherichia coli* strain in faeces, urine and mouth of a female dog (that had been medicated with several antibiotics for recurrent urinary tract infection), and on the owners’ faeces and in several locations within the domestic environment such as floor, dog’s food bowl and refrigerator door handle.

Although several reports have documented the presence of multidrug-resistant *S. aureus*, *E. coli* and *Enterococcus* spp. in dogs and cats [36,37,38,39,40,41,42], reliable quantitative data are needed to provide a better understanding of the dynamics of antimicrobial resistance transmission between household animals and humans. The assessment of risk factors that contribute to the dissemination of drug-resistant bacteria, or corresponding genetic elements, between pets and their owners is essential for the implementation of safe handling procedures of companion animals and prudent use of antimicrobial substances in veterinary medicine.

### 2.2. Production Animals

Over the last 50 years the increasing demand for livestock products and developments in breeding, nutrition and management practices led to modifications in animal production systems [43]. The current level of intensification of animal production systems leaves no tolerance for disease outbreaks in production animals. Thus, various antimicrobial drugs have been administered at sub-therapeutic levels aiming at disease prevention [44,45]. In addition, the manipulation of gut functions and microbial habitat of food producing animals with low levels of antimicrobials (feed additives) has been recognized as an important tool for improving growth performance and feed efficiency [46,47]. These practices provide favourable conditions for selection, persistence and spread of antimicrobial-resistant bacteria at the farm level [48,49,50,51]. Due to public health concerns, a much greater scrutiny is now focused on antibiotic use in food producing animals, especially for antimicrobial agents that have human analogues [52].

Historically, the assessment of the biological consequences of antimicrobial use in food animals was restricted to zoonotic enteropathogens (e.g., *Salmonella* spp., *Campylobacter jejuni*, *Listeria monocytogenes*, *Yersinia enterocolitica*). Field investigations have demonstrated that agricultural use of antimicrobial agents increases the likelihood that these bacterial pathogens (that have food animal reservoirs) will develop resistance or cross-resistance to drugs approved for use in human medicine [53,54].

Since the last decade of the 20th century, there has been an increasing awareness of the effects of antimicrobial drugs development of resistance in animal’s commensal flora, such as *E. coli* and *Enterococci*. The prevalence of antimicrobial resistance in the commensal bacteria (which are naturally occurring host flora) is an indicator of the selective pressure caused by the use of antimicrobial agents and reflects the potential for future resistance in future pathogens [55]. The levels of antibiotic resistance in *E. coli* and *Enterococci* have reached a point where they pose several clinical challenges to humans [31]. In many cases, the origin of the bacterial that cause infection in humans remains unknown, and the significance of the animal reservoir of antimicrobial-resistant *E. coli* and *Enterococci* has not been completely quantified. Nevertheless, it has been found that farm workers pick up resistant strains to specific agents given to animals [56] and resistant strains have been found on foodstuffs (meat, eggs and some dairy products) at points of sale [57,58,59]. In addition, as faecal waste from food animals is often spread as fertilizer, there is an ongoing environmental dissemination of strains that carry resistance to antimicrobial agents that are regarded as highly or critically important in human therapy [60,61]. This may threaten the clinical utility of some antimicrobial agents and, so far, has received little attention [62].

Paradoxically, the use of antimicrobial agents in modern animal husbandry has important resemblances with its use in human hospitals. In both ecological niches: (i) antimicrobial agents are heavily prescribed, (ii) decisions on drug use often rely on the risk of infection rather than on the existence of the infection itself, (iii) and a “resident microbiota” is exposed to a selective density due to the simultaneous/successive use of different antimicrobials. This practice creates special conditions for the selection, spread and evolution of resistant strains and the establishment of stable resistance traits [9,63,64,65,66].

Due to public health concerns, the addition of nontherapeutic antimicrobial drugs to animal feeds was banned by countries of the European Union in January 2006. In Denmark, all use of antimicrobial agents as growth promoters was voluntarily banned in 1986. This decision was followed by a decrease in antimicrobial-resistant bacteria in animals, food products, and humans [67,68]. However, European producers' expectations regarding performance enhancement and illness prevention have led to an increased use of therapeutically valuable agents [31]. Field investigations aiming to evaluate the selective impact on *E. coli* and *Enterococcus* spp. of the use of preventive antimicrobial drugs in poultry production, concluded that the selective pressure exerted by these drugs is impressive and cumulative [34,69,70]. Interestingly, antimicrobial resistance in *E. coli* was mainly medication-dependent, whereas among *Enterococci*, changes observed over time were apparently influenced by factors other than antimicrobial exposure, such as the drug-resistant bacteria previously present in the farm environment and those present in feedstuffs [70,71]. Previous studies have also supported the idea that farm indigenous microbiota and feed-associated bacteria may have a higher influence on the prevalence of antimicrobial resistance than antimicrobial usage itself [34,54]. In fact, ubiquitous microorganisms such as *E. coli* and *Enterococci*, which are very successful in adapting to different hosts and environments, increases the complexity of the role of the different evolutionary forces involved.

## 3. Environmental Dispersion of Antimicrobial Resistance

**Figure 2:**
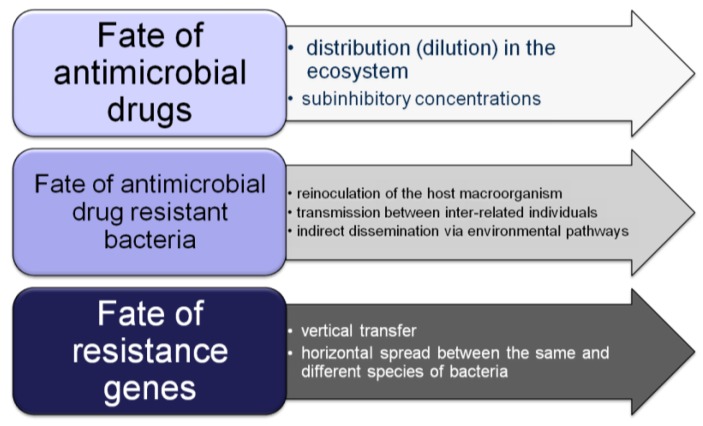
The post-antimicrobial effects.

The environment is the melting pot of antimicrobial resistance. Firstly, because antimicrobial resistance genes, that were acquired by bacterial pathogens, were imported from the environmental microbiota. The number of potential resistance genes present in the natural resistome is still far from being correctly estimated, and studies in this field are needed in order to understand the cycle of acquisition of these genes by human pathogens [72]. Secondly, for many pathogens of current concern, especially organisms for which asymptomatic colonization typically precedes infection, the selective effects of antibiotic use can only be understood if we consider the numerous environmental pathways (e.g., water, air, soil and mechanical vectors) that enable these bacteria, and the genes they carry, to spread between different biomes (Figure 2). However, it remains unclear why some bacterial lineages can spread rapidly whereas others that are equally resistant do not, posing challenges to the adoption of adequate measures for prevention and control of resistant strains. To answer this question, it is necessary to understand which factors among multidrug-resistant strains allow them to survive outside their hosts long enough to get an opportunity, even transitionally, to contaminate or colonise different hosts. The resistance of a particular strain to abiotic factors may be crucial to predict its ability to recolonize ancient hosts and to colonize new hosts. 

Effluents from urban areas and animal production units (husbandry and slaughter houses), even when treated in wastewater treatment plants, discharge resistant bacteria in the receiving surface waters [61,73,74,75,76]. Antimicrobial substances also can “survive” sewage treatment, being released into the soil and water [77,78,79]. These antibiotics are not readily biodegradable in sediments or in aquatic systems [80,81]. Thus, the emergence of a resistant pathogen could occur distantly from the original place where such drugs were prescribed and a long time after the original selection pressure [82]. In this scenario, the higher the amount of prescribed antibiotics, the higher will be its dispersion into the biosphere, increasing the chances of a complex microbial population present in a certain biome (e.g., river, lake or soil) to contact with the ideal selective concentration of those substances [7]. This ecological process has received only scant attention, when compared to the extensive work that has been carried on the effects of antimicrobial substances on individual pathogenic bacteria. Since those genes were not previously present in human pathogens, the only suitable source for them was the environmental microbiota [83,84].

The receiving surface water resources represent an important fraction of public drinking water, and standard treatment processes may not be completely efficient in removing or inactivating all infective organisms [27,85]. Furthermore, drug-resistant bacteria discharged into water bodies are ultimately transported to estuaries, river outlets and beaches [86,87].

The use of sludge on fields and pastures as a fertilizer represents another important pathway to the environment. From farmland soils, resistant bacteria could be dragged to ground and surface waters [88] or contaminate vegetables, when an insufficient period of time from fertilization-to-planting and fertilization-to-harvest is observed [89].

The ability of antimicrobial resistant strains to survive sewage treatment systems should be considered in the design of future plants to be built in a way that the number of resistant bacteria could be significantly reduced in both treated effluent and sludge, preventing their massive release into the environment. Unfortunately, the presence of selective antibiotic concentrations is a problem that is harder to solve, since there are no current technical solutions to eliminate them from sewage waters.

Biodiversity is an essential element in the equilibrium and regeneration of ecosystems [90]. It has been suggested that biodiversity loss may be the driving force for the emergence of infectious diseases [91]. Habitats with low biodiversity due to human interventions are less efficient in the interruption of the dissemination of infectious agents. Interestingly, this concept is extendable to antimicrobial resistance spread. The freshwater bivalve *Anodonta cygnea*, threatened with extinction due to fishing activities, extraction of inert materials and pollution, is a perfect example of counterintuitive allies in the reduction of environmental reservoirs of resistant bacteria. As filter feeders, bivalves are exposed to a constant challenge by various pathogenic bacteria when grown in polluted waters [92]. The persistence of these bacteria within bivalve tissues largely depends on their sensitivity to the bactericidal activity of the hemolymph [93,94]. In a study conducted by Antunes *et al*. [95], it was observed that the mean concentration of bacteria in hemolymph and extrapallial fluids of *A. cygnea* varied between 1.5 × 10^2^–6.5 × 10^2^ CFU·mL^−1^, and that *Vibrio metschnikovii* and *Aeromonas sobria* were the predominant species. However, *E. coli* and *Enterococci* were not detected in any of the healthy *A. cygnea* specimens, although these bacteria were found to be abundant in the water and mud of their habitat (a recipient of urban sewage water, surrounded by farms where fertilizers of animal origin were used). Surprisingly, examination of the hemolymph fluid of these bivalves revealed the presence of a large number of *E. coli* bacilli inside granulocytes suggesting that these freshwater mussels had the capacity to actively and specifically phagocyte *E. coli*. In an earlier investigation, Cavallo *et al*. [96] also suggested that the ability of the mediterranean mussel *Mytilus galloprovincialis* to filter and concentrate bacteria could contribute to the reduction of bacterial concentrations in seawater, thus playing an important role in the process of bioremediation of the marine environment. Besides the biologic importance of these observations, it is important to search for their ecological and medical relevance. From an ecological perspective, the ability of these bivalves to filter and eliminate bacteria is of utmost interest, as this could contribute to reduce the burden of unwanted microorganisms in aquatic ecosystems, reducing the probability of their re-introduction in human or animal populations. Medically, it will be important to identify the underlying immune mechanisms for the recognition, chemotaxis, attachment and destruction of antimicrobial-resistant *E. coli* and *Enterococcus* spp. strains.

## 4. Microbial Resistances in Wild Animals

The presence of multidrug-resistant bacteria has recently been reported in wild birds (gulls, birds of prey) and mammals (wolves, foxes, rabbits, deer, otters) with no apparent exposure to antimicrobials [97,98,99,100,101]. These findings suggest that resistance, once developed, is not confined to the limits of the ecological niche where it primarily emerged.

During the past decade, extended-spectrum β-lactamases (ESBL) have been identified in birds of prey [102,103]. Since none of the sampled birds had received antibiotics and few have been previously fed by humans, the question, once again, relies on the sources and pathways of their acquisition of these multidrug-resistant strains. Two hypothesis may justify the presence of these phenotypes in wild birds inhabiting scarcely populated regions: (i) colonization of the birds’ gut with resistant strains directly from the environment or harboured by their prey or, (ii) when colonization is not possible due to host specificity, the share of transferable genetic elements that code for resistance between “ingested” strains and the native enteric flora of wild birds. The latter occurs preferably between bacteria with the highest phylogenetic proximity, but it may also happen between different genus and species [13,14,104]. *E. coli* is known for its capacity to exchange resistance determinants between strains, particularly if plasmid-born [31]. In addition, we can not exclude that treated/contaminated preys or contaminated water may serve as a vector for residual quantities of antibiotics, high enough to pressure the emergence or the enrichment of resistant strains among the enteric flora of these birds. This has serious implications in the epidemiology of antimicrobial resistance both in humans and animals. Observations by Bonnedahl *et al*. [105] suggested wild birds could act as important environmental bio-indicators, and reservoirs, of medically important pathogens and of resistance genes, as well as a potential melting pot for the development of new resistance types.

Gulls have been identified as possible reservoirs for multidrug-resistant bacteria [105,106]. The Porto coastline in Portugal, including downtown Porto, has a large gull population (mainly *Larus fuscus* and *L. cachinnans*). Simões and co-workers [107] collected gull’s faeces from beaches, obtaining 139 *E. coli* isolates, of which 45 (32%) displayed an ESBL phenotype. Forty-four (98%) of the 45 ESBL producers carried a *bla*CTX-M gene. This study identified two unquestionable matters of concern for human health: that 37% of all ESBL isolates belonged to B2 or D phylogroups, which are related to extra-intestinal infections [108]; and that there was a high frequency of the determinant CTX-M-15 (34%). The latter finding points out to the spread of resistance from the “closed” environment of hospitals to the open environment, as CTX-M-15 are the most prevalent ESBL in *E. coli* in Porto’s hospitalized patients [109]. Hence, this numerous population of birds constitutes an important reservoir of ESBL strains, potentiating its “devolution” to the human population. This transmission is favoured by the near contact between these birds and humans (e.g., during beach recreational activities). Moreover, migratory gulls, such as *Larus fuscus*, crossing an extensive portion of the European coastline between the Mediterranean and Scandinavia, may be reservoirs for these emerging resistance determinants. The presence of multidrug-resistant strains in gulls demonstrates that the selective effects of antimicrobials are far beyond their prescription. In fact, the sewers where these birds search for food may provide ideal conditions for resistance emergence. The abundant and diverse microbiota, that hosts a large pool of resistant determinants, coupled with the presence of sub-inhibitory concentrations of antimicrobials (due to the elimination of these substances in faeces and urine of medicated individuals) allows the emergence of resistant clones and favours the transfer of genes between pre-existing resistant bacterial organisms and the susceptible ones [110].

More recently, several *E. coli* strains displaying an ESBL phenotype were found in faecal samples from Iberian wolves (*Canis lupus signatus*) [101], confirming, once more, the environmental dispersion of these phenotypes in the absence of a specific selective pressure. Several studies [98,100] have reported the presence of ESBL *E. coli* strains among natural preys of wolves (e.g., wild boars, rabbits and deer). Furthermore, as a consequence of the expansion of urban and agricultural areas, the feeding habits of many lupine populations have changed; it has been reported that although they ingest live domestic ungulates (mostly sheep and calves reared by extensive methods), they also have access to cadavers of animals from intensive farming production systems (mainly poultry and rabbits), that were left outside or only superficially buried [111]. The fact that wolves may become reservoirs of multidrug-resistant strains represents an environmental health hazard and, simultaneously, an increasing threat to the preservation of species, since many of these multidrug-resistant bacteria may harbour several virulence factors [112] that confer a higher capacity to colonize and cause disease to their animal hosts, particularly when they are immunosuppressed due to parasitic or viral infections (e.g., parvovirus) or nutritional deficiencies.

## 5. Conclusions

Antimicrobials are essential to save lives. Widespread concern has led to a plethora of governmental and agency reports advocating for less and better antimicrobial use, better infection control and the development of new drugs. However, as resistance to antimicrobials is becoming increasingly widespread without any plausible association with the use of these drugs, it is necessary to seriously consider other strategies in order to prevent the emergence and dissemination of antimicrobial resistant bacteria. These strategies require a more holistic and forward-looking approach that take the complex interconnections among species into full account, recognizing the important link between human and animal health in accordance with the Manhattan principles on “One World, One Health”. Strains with a wider ecological hardness have a near-universal advantage, enabling them to thrive in various interconnected ecological niches. The fact that resistant microorganisms can survive in a wide range of potential niches and adapt to alternative hosts is worrisome, and this could amplify their capability to acquire new determinants both in terms of virulence and resistance, while simultaneously maintaining their fitness. Thus, to avert the emergence of resistance, efforts should be aimed not only at reducing the amount of antimicrobials used but also the establishment of barriers to effectively prevent the contamination of the environment by these drugs and by multidrug-resistant strains. This could be achieved through the application of improved sanitary measures to contain resistant strains inside health care settings and animal production facilities and the development of new drugs to be co-administered with antimicrobials intending to reduce their collateral selective effects on the environmental microbiome, without impacting antimicrobial efficacy to cure the infections.
